# Supplementary material for: Content-rich biological network constructed by mining PubMed abstracts
Source: BMC Bioinformatics. 2004 Oct 8;5:147. doi: 10.1186/1471-2105-5-147 (PMC528731; doi:10.1186/1471-2105-5-147)
Supplement: Additional File 2 — The original results of the above study (non-essential files are deleted to keep the file size under the limit set by BMC bioinformatics). [file 1471-2105-5-147-S2.bz2 › chilibotAdditionalFile2/dip20/499ID7706250E10662/html/TGFB1_TGFBR2.html]

 


 **TGFB1** and **TGFBR2** 
  
Found 302 abstracts in PubMed, retrieved 20.  
 

 What does Google say? 
 PDF only 
| .edu only 

---

**Interactive relationship** (e.g. stimulation, inhibition, etc)

**Non-interactive relationship** (e.g. studied together, co-existance, homology, etc.)

- While many tumor cell lines no longer respond to the inhibitory effects of TGFB  [ **TGFB1** ]  due to a reduction or absence of the type II receptor  **TGFBR2** , the role of  **TGFBR2**  in tumors from patients with breast cancer is less clear.  Ref: 12825850 Breast Cancer Res Treat, 2003
- These mutations are similar to those of the 10 bp polyadenine tract within the TGF beta  [ **TGFB1** ]  type II receptor  **TGFBR2** , a well characterized target of frameshift mutations in the same neoplasms.  Ref: 12615714 Cancer Res, 2003
